# High strength nanostructured Al-based alloys through optimized processing of rapidly quenched amorphous precursors

**DOI:** 10.1038/s41598-018-19337-7

**Published:** 2018-01-18

**Authors:** Song-Yi Kim, Gwang-Yeob Lee, Gyu-Hyeon Park, Hyeon-Ah Kim, A-Young Lee, Sergio Scudino, Konda Gokuldoss Prashanth, Do-Hyang Kim, Jürgen Eckert, Min-Ha Lee

**Affiliations:** 1 Advanced Functional Materials R&D Group, Korea Institute of Industrial Technology, Incheon, 21999 Korea; 2 Advanced Analysis Center, Korea Institute of Science and Technology, Seoul, 02792 Korea; 3 Deparment of Advanced Materials Engineering, Yonsei University, Seoul, 03722 Korea; 4 IFW Dresden, Institute for Metallic Materials, Helmholtzstraße 20, D-01069 Dresden, Germany; 5 IFW Dresden, Institute for Complex Materials, Helmholtzstraße 20, D-01069 Dresden, Germany; 6 Erich Schmid Institute of Materials Science, Austrian Academy of Sciences, Jahnstraße 10, A-8700 Leoben, Austria; 7 Norwegian University of Science and Technology, Teknologivegen 22, 2815 Gjøvik, Norway; 8 Department Materials Physics, Montanuniversität Leoben, Jahnstraße 10, A-8700 Leoben, Austria

## Abstract

We report the methods increasing both strength and ductility of aluminum alloys transformed from amorphous precursor. The mechanical properties of bulk samples produced by spark-plasma sintering (SPS) of amorphous Al-Ni-Co-Dy powders at temperatures above 673 K are significantly enhanced by *in-situ* crystallization of nano-scale intermetallic compounds during the SPS process. The spark plasma sintered Al_84_Ni_7_Co_3_Dy_6_ bulk specimens exhibit 1433 MPa compressive yield strength and 1773 MPa maximum strength together with 5.6% plastic strain, respectively. The addition of Dy enhances the thermal stability of primary fcc Al in the amorphous Al-TM -RE alloy. The precipitation of intermetallic phases by crystallization of the remaining amorphous matrix plays important role to restrict the growth of the fcc Al phase and contributes to the improvement of the mechanical properties. Such fully crystalline nano- or ultrafine-scale Al-Ni-Co-Dy systems are considered promising for industrial application because their superior mechanical properties in terms of a combination of very high room temperature strength combined with good ductility.

## Introduction

Many studies have been performed for producing light weight and high strength alloys with the aim of developing new materials for high performance transport systems and energy conservation. In particular, high strength aluminum-based alloys are gradually substituting steel and cast iron in automotive and aerospace industries to reduce the weight of the vehicles in order to decrease fuel consumption^1,2^. Along this line, several methods have been tried such as heat treatment and grain refinement to improve the strength of aluminum alloys. Moreover, rapid solidification, mechanical alloying or high pressure deformation have been recently used to produce high strength aluminum alloys^3–5^. However, paradoxically the high strength aluminum alloys produced above techniques are not always favorable in engineering application because high strength means difficulty of formability^6^.

Amorphous alloys or metallic glasses are well known by extreme high strength at room temperature however interestingly they can be softened to viscous liquid states above glass transition temperature where thermoplastic forming can be carried out by superplasticity^7^. Al-based amorphous alloys have been studied because of their extraordinary weight to high strength compared to conventional crystalline light-weight Al-based alloys at room temperature^8–14^. The formation of Al-based amorphous alloys by liquid quenching was first investigated for binary Al-M (metalloid) and Al-TM (transition metal) systems with coexisting amorphous and crystalline phases^8^.

However, although Al-based amorphous alloys exhibit superior mechanical properties compared to conventional Al-based crystalline alloys, the maximum scale of the products is limited to a thickness of only a few micrometers (under millimeter) due to their relatively low glass-forming ability requiring, in turn, high cooling rates upon solidification^15^. Moreover, Al-based amorphous alloys deform by highly localized shear banding introduced a catastrophic failure which is similar to typical characteristic of amorphous alloys resulting in show little overall room temperature plasticity^16^. In these reason, the previous studies in Al-based amorphous alloys are focused on how to improve both glass forming ability and mechanical properties of amorphous alloys or bulk metallic glasses through processing and fabrication routes^15–18^.

In the subsequent development of Al-based high-strength nanocrystalline alloys, Wang *et al*. have presented enhanced mechanical properties of Al-Ni-Co-Gd by nanocrystallization of amorphous alloys^19^. Moreover, it has been presented that introducing additional rare metal alloying elements could promote the glass forming ability and modify the mechanical properties of metallic glasses^17,18^. Qiao *et al*. investigates that the micro-alloying of Dy could play an important role to influence plasticity of the Cu-based metallic glasses^17^. Park *et al*. shows that the addition of Y has been improved mechanical properties of the Cu-based metallic glasses by introduction of chemical inhomogeneity^18^. Inoue *et al*. have found that alloys comprised of an amorphous phase together with nanoscale Al phases in rapidly solidified Al_88_Ni_7_Co_5_ exhibit strengths that are superior to those of an amorphous single phase^20^. However, although partially or fully nano-crystallization of Al-based amorphous alloys exhibit overcoming of enhanced plasticity of the intrinsic brittleness of amorphous, the limitation of scale does not change resulting in very limited size. To overcome this drawback, powder metallurgy methods such as consolidation of gas-atomized amorphous powders have been used to surmount the size limitation^21–24^. These limitations have prevented widespread application of Al-based amorphous and partially (nano)-crystalline alloys even despite their excellent mechanical properties^25^.

In magnetic materials, Dy is critical element to achieve sustaining thermal stability of high performance Nd-based magnets. Moreover, the effect of each RE element, especially Dy, on the thermal stability of the amorphous phase and the correlation with the mechanical properties during crystallization is still unexplored. And the impact of the synthesis route on the crystallization kinetics for obtaining plasticity under such extremely high strength values in Al-based alloys is still unclear^26^.

In this study, we attempted to improve the thermal stability of Al-based amorphous alloys by introducing Dy, and to obtain enhanced mechanical properties for such Al-based alloys by *in-situ* nanocrystallization during sintering of amorphous precursor powders. Along this line, we systematically studied the crystallization kinetics as well as the phase and microstructure transformation of an amorphous Al_84_Ni_7_Co_3_Dy_6_ alloy, and correlate the findings with the mechanical properties and strengthening mechanism(s) of this model alloy.

## Results and Discussion

**Figure 1:**
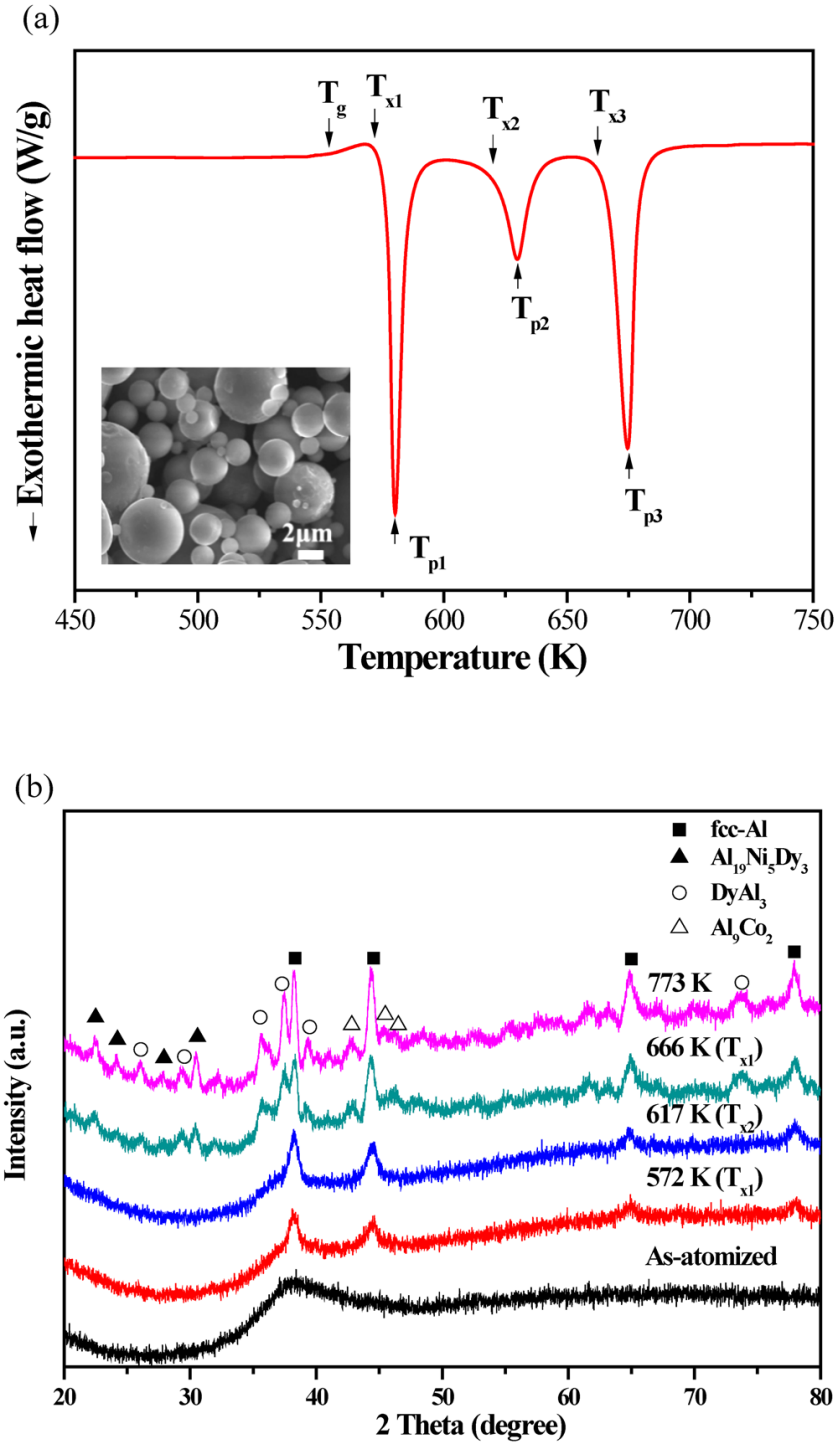
(**a**) DSC trace obtained during continuous heating of gas-atomized Al_84_Ni_7_Co_3_Dy_6_ powder at a heating rate of 40 K/min (inset image shows morphology of powder); (**b**) XRD patterns of gas-atomized Al_84_Ni_7_Co_3_Dy_6_ powder heated to 572 K, 617 K, 666 K and 773 K, respectively.

Figure 1(a) shows typical DSC traces obtained during continuous heating at a heating rate of 40 K/min for the gas-atomized Al_84_Ni_7_Co_3_Dy_6_ amorphous powder with a particle size below 25 µm diameter. The DSC trace of the amorphous Al-Ni-Co-Dy powder exhibits an endothermic event corresponding to the glass transition into the supercooled liquid at 559 ± 1 K. The supercooled liquid region Δ*T*_x_ = *T*_x1_−*T*_g_, is about 17 K. Three exothermic peaks corresponding to sequential crystallization of the supercooled liquid and phase transformation into crystalline compounds occur at onset temperatures of *T*_x1_ = 570 ± 1 K, *T*_x2_ = 620 ± 1 K and *T*_x3_ = 663 ± 1 K. The peak temperatures of the exothermic crystallization are *T*_p1_ = 580 ± 1 K, *T*_p1_ = 630 ± 1 K and *T*_p1_ = 675 ± 1 K, respectively. These *T*_g_, *T*_x_ and *T*_p_ values of the Al-Ni-Co-Dy amorphous powder agree well with the values obtained for the melt spun Al-Ni-Co-Dy amorphous ribbons (shown in Supplementary S1). The enthalpies of crystallization, ∆*H*, related to the exothermic DSC peaks are ∆*H*_1_ = −41 ± 0.5 J/g, ∆*H*_2_ = −25 ± 0.5 J/g and ∆*H*_3_ = −50 ± 0.5 J/g, respectively. The thermal properties of the amorphous Al_84_Ni_7_Co_3_Dy_6_ powder are summarized in Table 1.

**Table 1:** Characteristic thermal stability data (heating rate 40 K/min) of amorphous Al_84_Ni_7_Co_3_Dy_6_ gas-atomized powder.

Contents	T_g_(K)	T_p1_(K)	T_p2_(K)	T_p3_(K)	ΔT(K)	ΔH_1_(J/g)	ΔH_2_(J/g)	ΔH_3_(J/g)	Total ΔH (J/g)
Al_84_Ni_7_Co_3_Dy_6_ powder (<25 µm)	559 ± 1	580 ± 1	630 ± 1	675 ± 1	17 ± 1	41.3 ± 0.5	25.0 ± 0.5	50.3 ± 0.5	116.6 ± 0.5

Figure 1(b) shows the XRD patterns obtained for heat treated Al_84_Ni_7_Co_3_Dy_6_ powder. The XRD pattern for the as-atomized powder shows broad diffraction maxima, characteristic of the amorphous structure. In order to study the structural evolution upon heat treatment, the amorphous powder was constant-rate heated in the DSC by at a continuous heating rate of 40 K/min. The amorphous Al-Ni-Co-Dy powder, heated up to the first exothermic reaction temperature (*T*_x-ann1_ = 572 K), shows sharp diffraction peaks due to the crystallization of α-Al. When the sample is heated up to the third exothermic reaction temperature (*T*_x-ann3_ = 666 K), the XRD pattern shows the diffraction peaks from α-Al, Al_19_Ni_5_Dy_3_ (Pearson symbol oC108, space group Cmcm), DyAl_3_ and Al_9_Co_2_ intermetallic phases together with a weak diffuse background, respectively. Comparing DSC and XRD results reveals that the crystallization sequence of the amorphous Al-Ni-Co-Dy powder is identical to the crystallization behavior of the amorphous Al-Ni-Co-Dy ribbons (shown in Supplementary S1).

**Figure 2:**
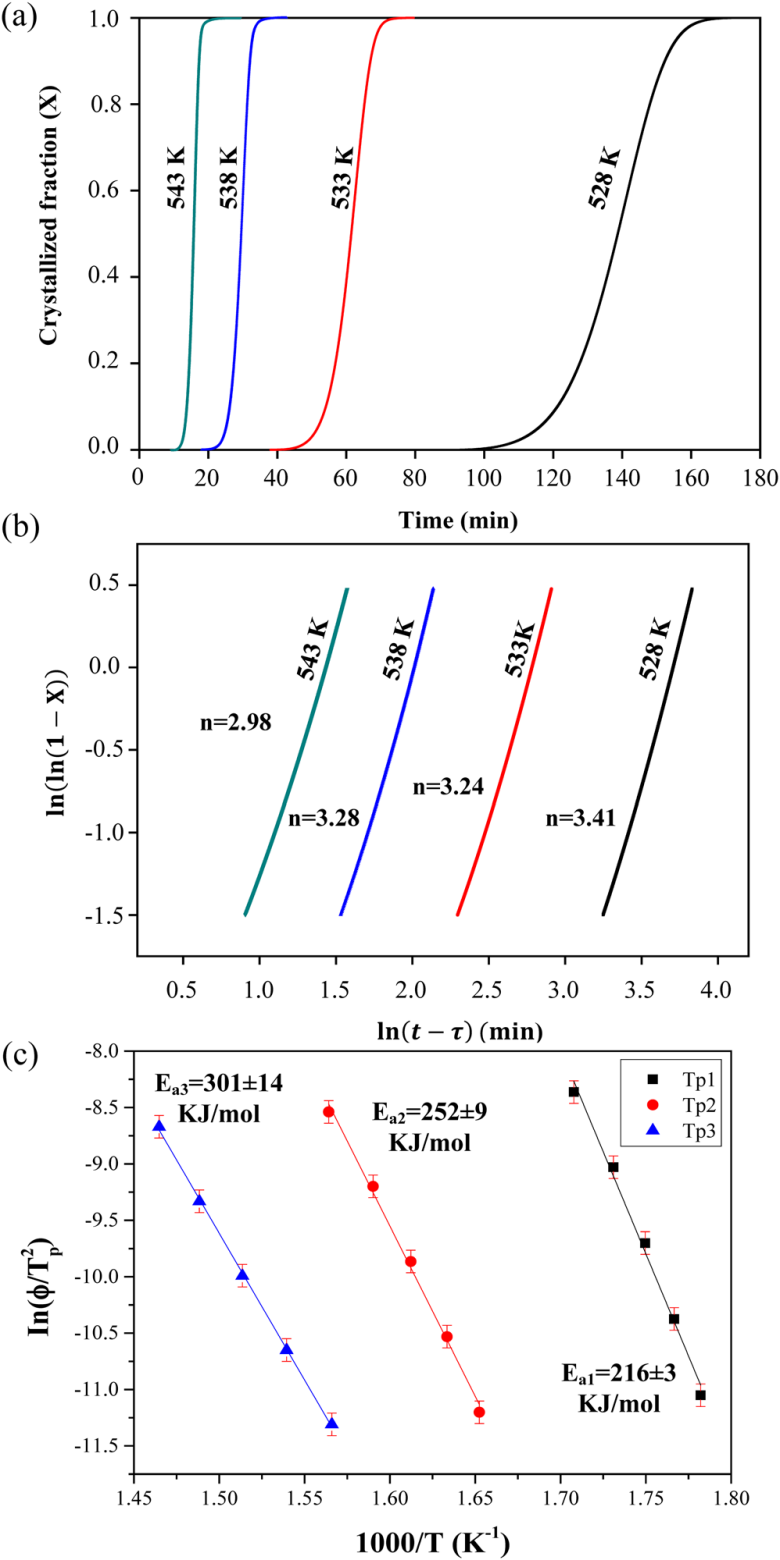
(**a**) Crystallized volume fraction (*X*) vs. time (*t*) relationship; (**b**) ln[ln{1/(1-*X*)}] vs. ln(*t*-*τ*) plots for different annealing temperatures for the Al_84_Ni_7_Co_3_Dy_6_ amorphous alloys. (**c**) Activation energy (*E*_*a*_) for crystallization of the Al_84_Ni_7_Co_3_Dy_6_ amorphous alloys.

In order to generate bulk specimens and for subsequent investigation of their mechanical properties, the gas-atomized Al_84_Ni_7_Co_3_Dy_6_ powder was consolidated by spark plasma sintering (SPS). To assure that the resulting bulk specimens contain the desired phases and microstructures, it is necessary to check their crystallization behaviors and phase transformations at different temperature ranges during SPS processing. The kinetic behavior of the transformation from the amorphous phase/supercooled liquid to the first crystallized phase (α-Al) was studied further by isothermal and isochronal DSC experiments. The isothermal DSC traces were analyzed by the JMA equation^27–29^,

1 $$X=1-\exp (-X^{\prime} )=1-\exp (-k{t}^{n}),\,\ldots $$

where *X* is the volume fraction of the crystallized phase, *k* is the kinetic constant which depends on the annealing temperature *T*, and *n* is the Avrami exponent which depends on the crystallization mechanism. The fraction *X* of crystallized phase at time *t* during isothermal annealing can be expressed as,

2 $$\mathrm{ln}\,[-\mathrm{ln}(1-X)]=n\,\mathrm{ln}\,k+n\,\mathrm{ln}(t-\tau ),\,\ldots $$

where *τ* is the incubation time. The crystallized volume fraction (*X*) vs. time (*t*) relationship for melt-spun amorphous Al_84_Ni_7_Co_3_Dy_6_ ribbon annealed at different temperatures is shown in Fig. 2(a).

Figure 2(b) presents Avrami plots by plotting *ln[−ln(1−X)]* against *ln(t-τ)* for different annealing temperatures (0.2 < *X* < 0.8). The Avrami exponents (*n*), which provides information about the dimensionality of the transformation for different nucleation and growth mechanisms^30^, can be obtained from the slope of the straight line shown in Fig. 2(b). The average value of *n* for the first crystallization step in Fig. 2(b) is 3.22 ± 0.3 which indicates that the first crystallization stage of the Al_84_Ni_7_Co_3_Dy_6_ amorphous ribbon is a volume nucleation and two-dimensional growth process^31^. Similar events have been observed in other Al-RE-TM metallic glasses such as Al_86_Ni_6_Y_44.5_Co_2_La_1.5_ or Al_88_Gd_6_La_2_Ni_4_^32–34^. Meanwhile, the *n* values of the Al_84_Ni_7_Co_3_Dy_6_ samples vary from 2.98 ± 0.3 to 3.41 ± 0.3, for different annealing temperatures which means that the nucleation and subsequent growth of spherical crystals occurs in a three-dimensional mode inside of the bulk. The decrease of the Avrami exponent (*n*) from 3.41 ± 0.3 to 2.98 ± 0.3 with increasing annealing temperature can be explained from the phase formation of α-Al. When fcc Al nanocrystals are formed from the amorphous phase, they reject solute elements such as Ni, Co and Dy into the residual amorphous matrix^35^. This reduces the driving force for the formation of additional fcc Al and accumulates Dy around the α-Al precipitates, thus impeding diffusion of the elements required for further growth of the nanocrystals. The activation energy (*E*_*a*_) for crystallization of the Al_84_Ni_7_Co_3_Dy_6_ amorphous alloys can be estimated using the Kissinger method^36^, which describes the dependence of the crystallization peak temperature (*T*_*p*_) on the heating rate (*Φ*) by the following equation:^36^

3 $$\mathrm{ln}(\frac{{{\Phi }}^{{\rm{n}}}}{{{\rm{T}}}_{{\rm{p}}}^{2}})=\,-(\frac{m{E}_{{\rm{a}}}}{R{T}_{{\rm{p}}}})+\,\mathrm{ln}\,K,\,\ldots $$

where *R* is the gas constant, *K* is a constant containing factor depending on the thermal history of the sample, and *n* and *m* are constants depending on the morphology of the growth. As shown in Fig. 2(c), the slope of the straight line when plotting *ln(Φ/T*_*p*_^2^) versus *(1/T*_*p*_) represents *E*_a_/*R*, where *E*_a_ is the activation energy of crystallization obtained from the isochronal DSC traces of the Al_84_Ni_7_Co_3_Dy_6_ amorphous alloys recorded at different heating rates from 5 to 80 K/min. The calculated activation energies for each crystallization stage are *E*_a3_ = 301 ± 14 kJ/mol, *E*_a2_ = 252 ± 9 kJ/mol and *E*_a1_ = 216 ± 3 kJ/mol. The activation energy for the third crystallization (*E*_a3_ = 301 ± 14 kJ/mol) estimated by the Kissinger method shows a difference compared to the activation energy (*E*_a_ = 362 ± 24 kJ/mol) calculated by the JMA equation due to the overlapping with the growth of the first crystallization event and the nucleation of the second crystallization step.

**Figure 3:**
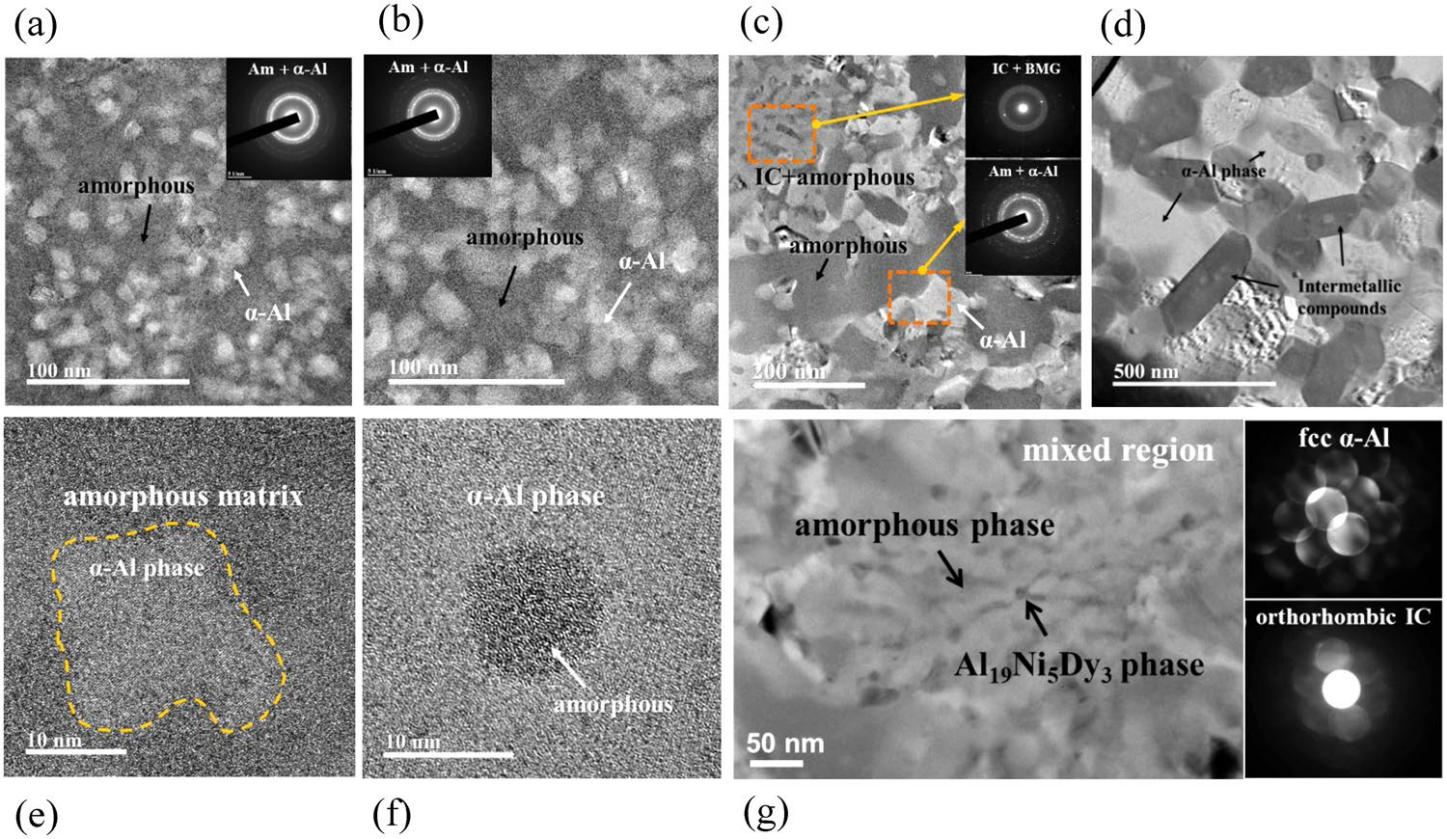
Bright field (BF) and high-resolution (HR-)TEM images and SAED patterns of the as-spun Al_84_Ni_7_Co_3_Dy_6_ ribbon annealed at (**a**) 601 K, (**b**) 646 K, (**c**) 667 K, and (**d**) 773 K; (**e**) high resolution HR-TEM image of the ribbon annealed at 646 K; (**f**) HR-TEM image of the amorphous phase mixed with α-Al after annealed at 667 K; (**g**) HR-TEM image of the amorphous phase mixed with orthorhombic Al_19_Ni_5_Dy_3_ phase as found for the ribbon annealed at 667 K, corresponding to the orange square dotted regions in (**c**).

Figure 3(a–d) show typical bright field (BF) TEM images obtained for Al_84_Ni_7_Co_3_Dy_6_ amorphous ribbons which were heated up to the exothermic reaction temperatures 601 K, (b) 646 K, (c) 667 K and (d) 773 K, respectively. The insets show the corresponding selected area diffraction patterns (SADP). The BF TEM images show a homogeneous distribution of the fcc α-Al phase dispersed in the amorphous matrix. The mixtures of crystalline and amorphous phases are also revealed in the inset SADPs of Fig. 3(a–c) which show sharp crystalline spots superimposed on the diffuse halo rings of the amorphous phase. Figure 3(d) shows a BF TEM image obtained from an amorphous ribbon annealed at 773 K, i.e. in the fully crystallized state. Rod-shaped intermetallic compounds with ~100 nm width and 200 ~ 300 nm length can be observed that are intermixed with α-Al phase, and there is a constrained growth of α-Al. These results are in agreement with the XRD results shown in Fig. 1(b).

Figure 3(e) displays a high resolution (HR) TEM micrograph of a ribbon annealed at 646 K. The crystalline phase identified as α-Al phase is homogeneously distributed with a volume fraction of ~63 vol.% and a size of 15 ± 4 nm. As shown in Fig. 3(c), when the heat-treatment temperature increases to 667 K, corresponding to *T*_p2_ in the exothermic heat flow curve, the size and volume fraction of the α-Al phase slightly increase to ~67 vol.% and a grain size of 50 ± 4 nm, respectively. However, even at this temperature a residual amorphous phase with ~10 nm size still remains besides the α-Al phase produced by crystallization [Fig. 3(f)]. Figure 3(g) shows a HR TEM image of the orange square dotted regions in (c), revealing the presence of amorphous phase mixed with orthorhombic Al_19_Ni_5_Dy_3_ phase for the ribbon annealed at 667 K. The nano-beam diffraction patterns (NBDPs) in Fig. 3(g) correspond to the [011] zone of the fcc Al phase and the [031] zone of the orthorhombic Al_19_Ni_5_Dy_3_ phase, respectively. The growth of the α-Al phase is constrained by crystallization of intermetallic compounds (IC) resulting in a relatively small size of α-Al (~50 nm) and the intermetallic phase (~10 nm). This limited growth of the α-Al phase together with the absence of other phase(s)/intermetallic compound(s) is consistent with observations reported previously for other Al-based metallic glasses^37,38^. The reason for this feature is attributed to solute enrichment in the matrix due to the precipitation of α-Al, even though α-Al is softer^39–41^.

**Figure 4:**
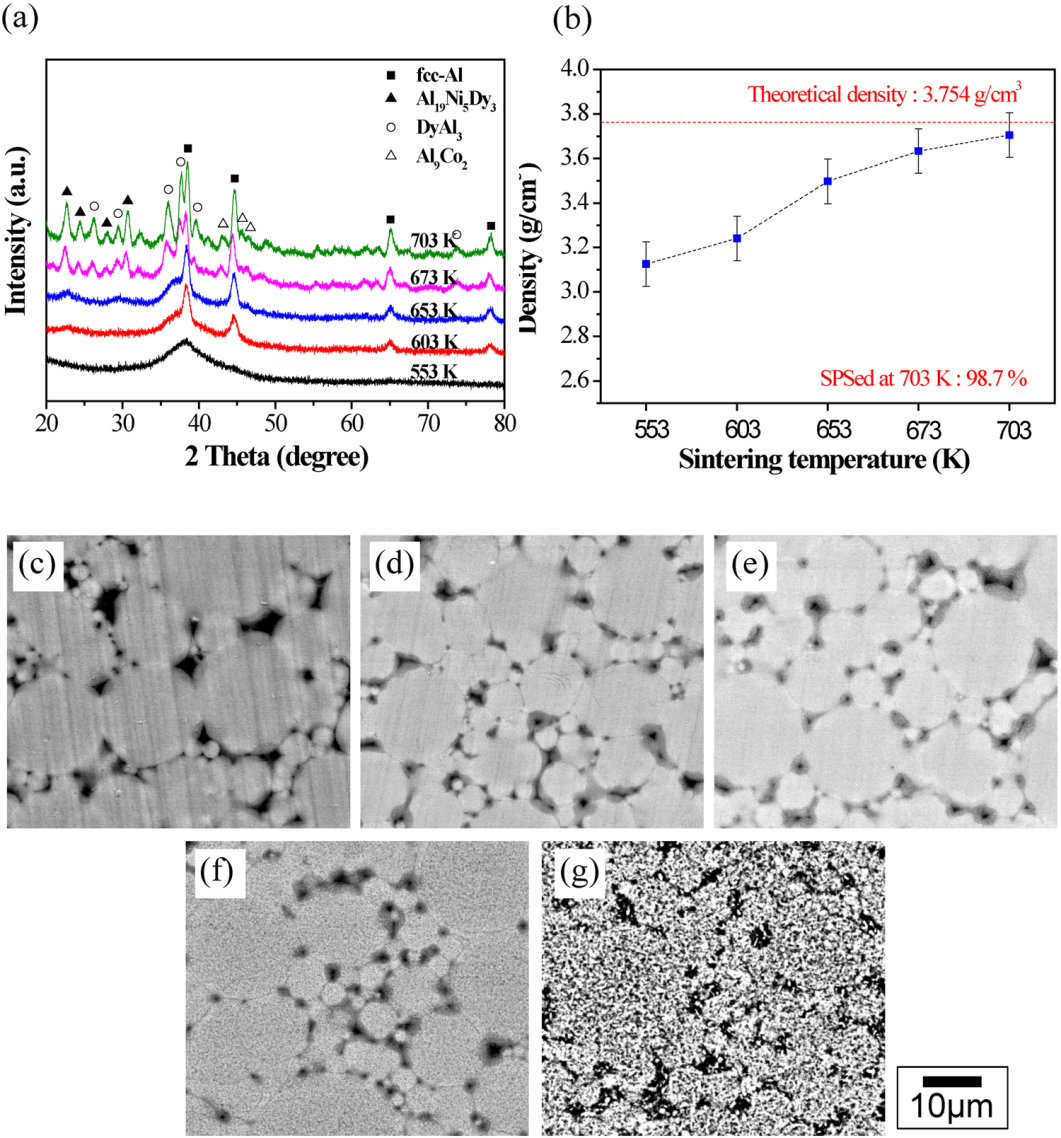
(**a**) XRD patterns of SPSed Al_84_Ni_7_Co_3_Dy_6_ samples subjected to different sintering temperatures; (**b**) sintered densities as a function of SPS sintering temperature; (**c**) to (**g**) are typical BSE mode SEM microstructures corresponding to the sintered samples at processed at the different temperatures shown in (**a**): (**c**) 553 K, (**d**) 603 K, (**e**) 653 K, (**f**) 673 K and (**g**) 703 K, respectively.

The SPS method not only provides thermal energy and pressure, but also electric energy within very short time^42–44^. The electric energy can penetrate the powder particles at their outer surface and leads to localized melting^42–44^. Figure 4(a) compares the XRD patterns of Al_84_Ni_7_Co_3_Dy_6_ bulk samples SPSed at different sintering temperatures between 553 K and 703 K under a fixed applied pressure of 500 MPa. The XRD pattern of the sample sintered at 553 K shows broad diffuse diffraction maxima similar to the XRD pattern of as-atomized powder [Fig. 1(b)], suggesting that the amorphous state is retained without crystallization at low SPS temperature. However, the increase in the sintering temperature from 553 K to 653 K leads to noticeable sharp diffraction peaks due to crystallization of the α-Al phase superimposed on the diffuse diffraction background of the amorphous matrix. With the sintering temperature increases to 673 K~703 K, the diffuse diffraction background disappears and additional diffraction peaks become visible that correspond to the crystallization of the residual amorphous matrix into Al_19_Ni_5_Dy_3_, DyAl_3_, and Al_9_Co_2_ intermetallic compounds. The measured densities of the bulk samples sintered at different temperatures are shown in Fig. 4(b). As expected, the density of the sintered compacts increases with increasing temperature. By the sintering temperature increases to 703 K, the density of the SPSed bulk samples reaches 98.7% of the theoretical density (=3.754 g/cm^3^). Fig. 4(c)–(g) show SEM images obtained from the bulk samples sintered at the different temperatures from 553 K to 703 K, respectively. As shown in Fig. 4(c–e), the microstructures of the samples sintered at temperatures between 553 K and 653 K exhibit a rather featureless contrast corresponding to the amorphous phase with some pores between powder boundaries. However, when the sintering temperature increases above 673 K small features with dark contrast corresponding to intermetallic compounds can be observed [Fig. 4(f) and (g)]. The size and the volume fraction of these intermetallics increase with increasing sintering temperature, as shown in Fig. 4(g). The inter-particle regions which are mainly soft α-Al with few pores are readily visible in the sintered samples. Wang *et al*. present that these inter-particle regions are crucial to enhance plasticity by branching and deflection of crack propagation^19,45^.

**Figure 5:**
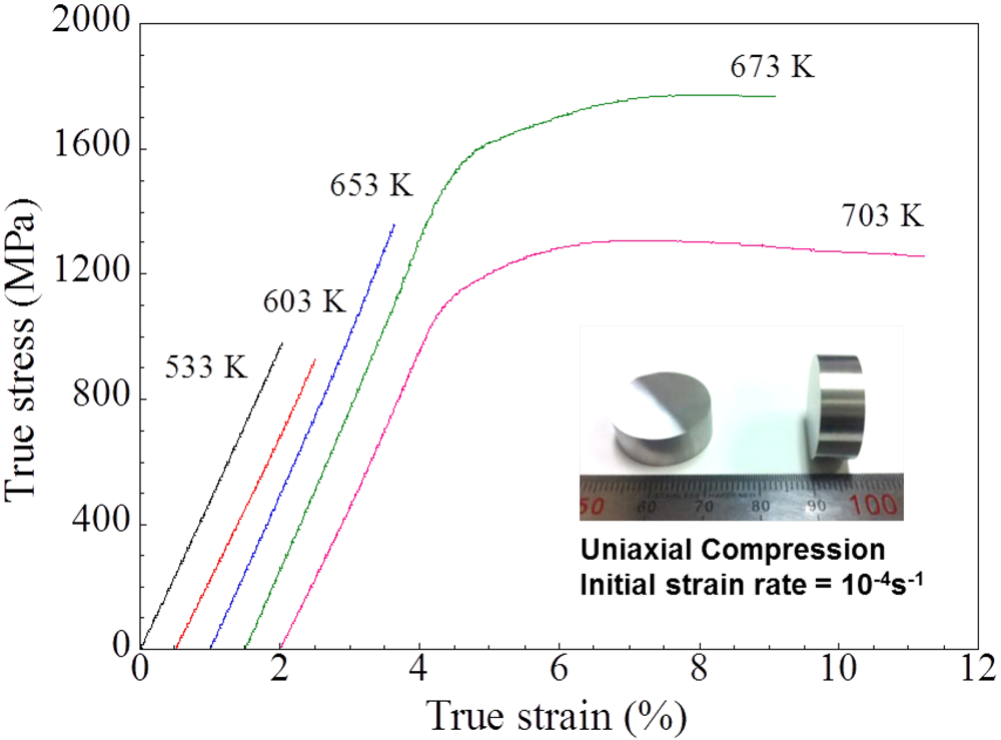
Room temperature true stress-strain curves of SPSed Al_84_Ni_7_Co_3_Dy_6_ bulk samples processed at different sintering temperatures; the inset image shows typical SPSed Al_84_Ni_7_Co_3_Dy_6_ bulk samples.

Figure 5 shows compressive true stress-strain curves of the SPSed Al_84_Ni_7_Co_3_Dy_6_ bulk samples fabricated at different sintering temperatures from 553 K to 703 K. The mechanical properties of the SPSed Al_84_Ni_7_Co_3_Dy_6_ samples are summarized in Table 2. The samples sintered at 673 K exhibit the highest yield strength and maximum strength about 1433 MPa and 1773 MPa with 5.6% of plastic strain, respectively. The plastic strain of the samples sintered at 703 K increases up to 7.2%, but the yield strength and maximum strength drop to 1068 MPa and 1255 MPa, respectively. The yield strength is lowered to 926 MPa and there is no discernible plasticity for the samples sintered at 603 K. The absence of plasticity is comparable with the findings for typical Al-based metallic glasses^46,47^ and nanocrystalline alloys^48^. The tendency of improving the strength by increasing the sintering temperature from 603 K to 673 K corresponds to more pronounced crystallization of α-Al phase, which shows matched results to the crystallization behavior and structural analyses during heating of Al_84_Ni_7_Co_3_Dy_6_ amorphous powders, as shown in Fig. 1 DSC trace and XRD patterns, respectively. The strength increasing through multiphase crystallization in Al-based metallic glass can be explained by composite effect of refined microstructures^19^. Kim *et al*. reported that the maximum strength could be obtained at the around 20~30% vol.% of nanocrystalline fcc-Al and the maximum strain reached at 20 vol.% in AlYNiFe amorphous alloy^49^. When the SPS temperature of Al_84_Ni_7_Co_3_Dy_6_ amorphous powder is relatively low temperature (~653 K) the strength and strain increase as increase volume fraction of nanocrystalline fcc Al (~63%). However, although ductile α-Al phase is present below 673 K, the size and volume fraction of this phase are not sufficient to improve the plastic deformability of the composites containing an amorphous phase. When the SPS temperature increased to ~673 K, the crystallization of nano-size intermetallic phases (around 10 nm) is mainly contributed to the strength enhancement.

**Table 2:** Mechanical properties of SPSed Al_84_Ni_7_Co_3_Dy_6_ bulk samples sintered at different temperatures.

Sintering temperature (K)	553	603	653	673	703
Yield stress (MPa)	979 ± 2	926 ± 2	1357 ± ± 2	1433 ± 2	1068 ± 2
Maximum stress (MPa)	979 ± 2	926 ± 2	1357 ± 2	1773 ± 2	1255 ± 2
Plastic strain (%)	—	—	—	5.6 ± 0.2	7.2 ± 0.2

Furthermore, the increase in plastic strain above 673 K with further increasing sintering temperature corresponds to increase size and volume fraction of intermetallic compounds (Al_19_Ni_5_Dy_3_, DyAl_3_, and Al_9_Co_2_) by more pronounced crystallization, as shown in Fig. 4(a). Meyers *et al*. presented that the strength of nanocrystalline (or ultrafine grained) metals (Cu, Fe, Ni and Ti) can be reached maximum value with ~80 nm crystalline size at compressive deformation^50^. Moreover, the enhanced plastic strain was observed at ~20 nm grain or crystalline size by the grain boundary sliding effect. It could be explained that the both growth of fcc-Al (~70 vol.%) nanoscrystals and initial stage of crystallization of intermetallic phases (~30 vol.%) can be contributed to enhanced plasticity above 673 K sintering temperature.

**Figure 6:**
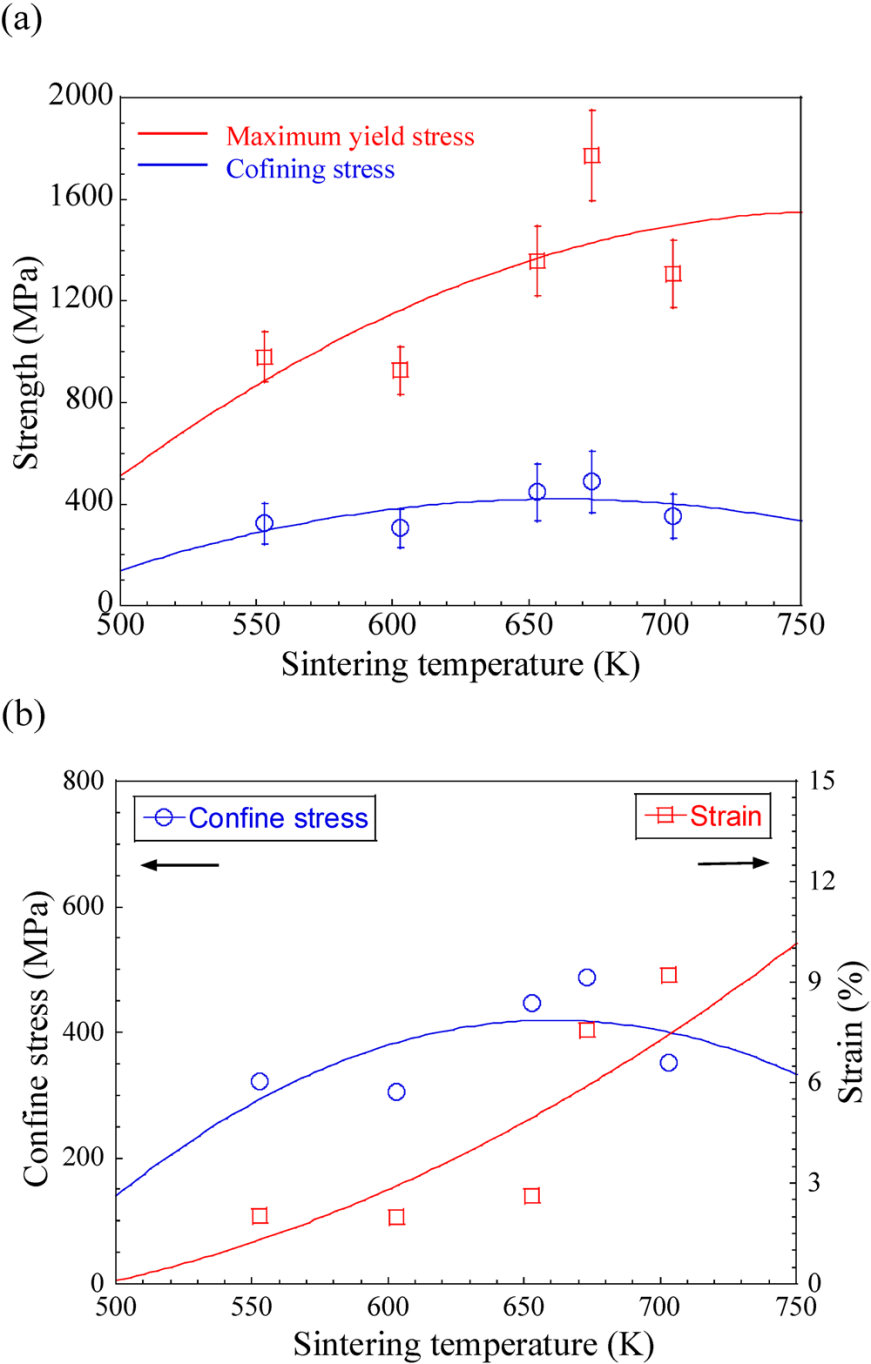
(**a**) Relationship between compressive maximum yield stress and confining stress; (**b**) relationship between plastic strain and confining stress in SPSed Al_84_Ni_7_Co_3_Dy_6_ bulk samples.

This enhanced plasticity by multiphase crystallization can be also considered by internal confinement effect among nanocrystalline phases^19,45^. Usually, the strengths of brittle materials, such as MGs, nanocrystalline materials and intermetallics, are sensitive to confined pressure, and difference of a tension-compression strength asymmetry can be analyses by the fracture strength under confining stress states. At room temperature quasi-static loading condition, the confining effect on the strength is related to compressive stress and the fracture criterion for compression can be described as^19^,

4 $${({\sigma }_{1}-{\sigma }_{3})}^{2}=2(2+{\alpha }^{2}){\tau }_{0}^{2}+2{\alpha }^{2}{\sigma }_{1}{\sigma }_{3},\,\ldots \,$$

where *α* is materials constant, *τ*_0_ is critical shear fracture stress, *σ*_1_ and *σ*_3_ are the maximum and minimum principal stresses, respectively (*σ*_1_ + *σ*_3_ < 0 for compressive loading condition). Since *τ*_*max*_ = (*σ*_1_ − *σ*_3_)/2, the effective shear yield stress, *τ*_*y*_, for compression can be also derived as^6,19^,

5 $${\tau }_{y}=\sqrt{\frac{(2+{\alpha }^{2}){\tau }_{0}^{2}}{2}+\frac{{\alpha }^{2}{\sigma }_{1}{\sigma }_{3}}{2}},\,\ldots $$

where $\sqrt{{\sigma }_{1}{\sigma }_{3}}\,\le ({\sigma }_{1}+{\sigma }_{3})/2$, one can define $\sqrt{{\sigma }_{1}{\sigma }_{3}}$ as the confining stress to characterize the magnitude of confinement. The relationship between maximum yield stress and confining stress was presented in Fig. 6(a), the yield stress increases as increase confining stress. For brittle materials, such as intermetallics or MGs, have a relative large *α* means the confinement is significantly influence the strength of materials. Wang *et al*. evaluated that confining stress introduced from the inter-confinement with well bonded interfaces could be endorsed additional strength enhancement eventually increasing overall strength^19^. The internal confinement could effectively prevent the brittle intermetallics and nanocrystalline fcc-Al from premature fracture and thereby provides an opportunity for plastic deformation^19,45^. In the current study, the plastic strain of sintered samples increases as the confining stress reached maximum level, as shown in Fig. 6(b). Therefore, internal confining stress during compressive loading also becomes key roles in enhancing plasticity by suppressing the initiation fracture of brittle phases, such as intermetallics, and increase shear resistance of Al-based nanocrystalline alloys.

**Figure 7:**
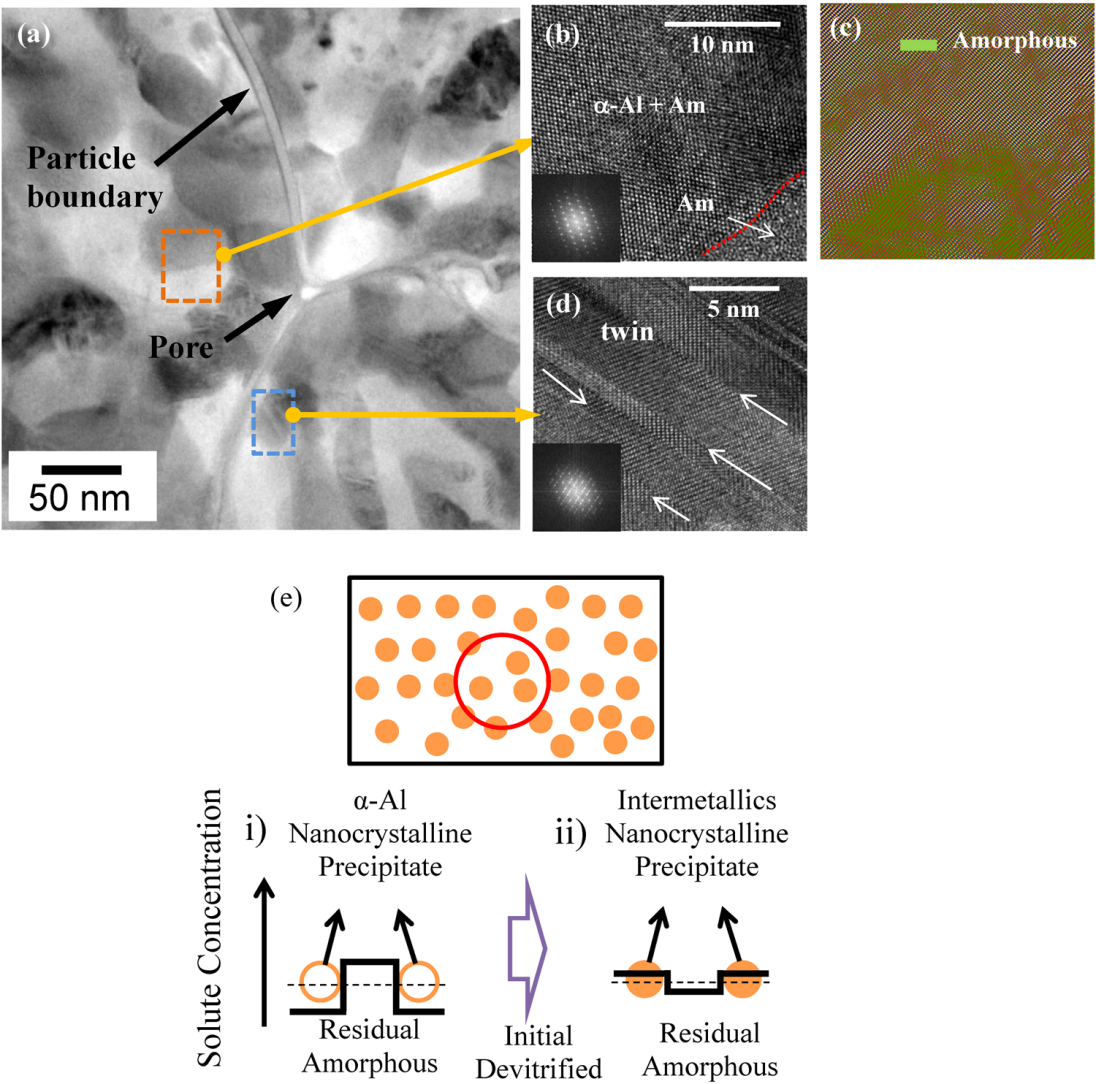
(**a**) Bright field (BF) TEM image of the SPSed Al_84_Ni_7_Co_3_Dy_6_ bulk sample sintered at 673 K; (**b**) HR-TEM image of the amorphous phase mixed with α-Al corresponding to the orange rectangular dotted region in (**a**); (**c**) Fast Fourier Transformation (FFT) of the HR-TEM image (**b**) revealing the distribution of the non-directional amorphous phase (green colored region); (**d**) twins of fcc α-Al phase formed by constraint growth corresponding to the blue rectangular dotted region in (**a**); (**e**) schematic model for the change of the concentration profile of solute atoms in the nanoscale particles embedded in the amorphous matrix during crystallization by heat treatment.

The internal confinement of the inside sintered powders can be verified by the observation of nanoscale twins in fcc-Al, as shown in Fig. 7(a) and (d) TEM images, which is known very hard to observed in fcc-Al even at high strain rate deformations due to their high stacking fault energy^51^. It was investigated that the effect of geometrically constrained stress-strain condition on the enhancement of mechanical properties with the formation of nano-twins in α-brass^52^. Figure 7 shows a BF TEM image [Fig. 7(a)] and a HR TEM image [Fig. 7(b)] for a bulk Al_84_Ni_7_Co_3_Dy_6_ sample obtained by SPS of the gas-atomized powder at 673 K. The BF TEM image in Fig. 7(a) exhibits a mixture of intermetallic compounds with gray contrast, some residual amorphous phase with dark contrast, and α-Al phase with bright contrast, respectively. Furthermore, Fig. 7(a) reveals particle boundaries, pores and the mixture of the phases. Inter-particle boundary regions show α-Al phase with bright contrast which is softer and more ductile than nanocrystalline inner-particle regions. Lee *et al*. evaluated that a deviation from a simple uniaxial state to a multi-axial stress state by geometrically constrained conditions in the uniaxial compressive loading sample^52^. It was presented that although the pores in the triple points can be the crack initiation points, locally high stress at the crack tip is relieved by the soft α-Al in the inert-particle regions in sintered Al_84_Ni_7_Gd_6_Co_3_ alloy^45,53^. Therefore, crack branching or deflection take place at the triple points by α-Al in the inert-particle regions act as sink for crack resulting in toughening the alloys. The HR TEM image in Fig. 7(b) was taken from the area marked by the orange dotted square in Fig. 7(a), and the coexistence of amorphous and α-Al phases, the inset SADP taken from α-Al phase shows fcc structure. An unmasked Fast Fourier Transformation (FFT) image is displayed in Fig. 7(c). It reveals the distribution of the non-directional (amorphous) phase (green colored region). Twins in the fcc α-Al phase that have formed due to constrained growth are shown in Fig. 7(d) HR TEM image and corresponding inset SADP, respectively, which was taken at the area marked by the blue rectangular dotted line in Fig. 7(a). These findings are in agreement with the XRD results shown in Fig. 4(a), and EDX analysis also reveals that the white and black regions in Fig. 4(f) consist of mostly Al and the content of Dy in the dark-gray regions is relatively high (4.88 at.% in white region and 6.39 at.% in dark region, respectively). Figure 7(e) shows a schematic of the composition changes of the partially devitrified alloy after heat treatment of the initially fully amorphous material. The devitrification process can be classified into different stages: i) solute enrichment of the system, ii) solute dilution according to the variations in the solute concentration during heat treatment^45,54–56^. The solute content of the amorphous matrix increases as the solute content decreasing within crystalline phases when the concentration of the precipitate is lower than the concentration of the initial amorphous alloy by crystallization of α-Al. On the contrary, the solute concentration of the amorphous matrix decreases with increasing concentration of precipitates by crystallization of intermetallic phases. As a result of the confined phase mixture models with the solute concentration redistribution model, progressively devitrified amorphous nanocomposites with fine nanoscale precipitate particles embedded in the amorphous matrix have been represented the strengthening mechanisms in Al_84_Ni_7_Co_3_Dy_6_ alloys.

## Conclusions

To summarize the relation between the mechanical properties and the nano-structural evolution of the Al_86_Ni_7_Co_3_Dy_6_ amorphous samples, it is believed that the precipitation and growth restriction of the α-Al phase are decisive factors to improve both the strength and the plasticity of the alloy. The mechanical properties of bulk samples produced from SPSed amorphous Al-Ni-Co-Dy powder at temperatures above 673 K are significantly enhanced by *in-situ* crystallization of nano-scale intermetallic compounds during the SPS process. The phase stability of devitrified Al_84_Ni_7_Co_3_Dy_6_ amorphous alloys has been successfully controlled by *in-situ* crystallization during SPS sintering. The decrease of the Avrami exponent (*n*) from 3.41 ± 0.3 to 2.98 ± 0.3 with increasing annealing temperature can be explained from the phase formation of α-Al and the first, second crystallization events of the Al_84_Ni_7_Co_3_Dy_6_ amorphous phase correspond to nucleation and two-/three- dimensional growth of fcc α-Al phase, respectively. The third crystallization stage is correlated with the phase transformation of the residual amorphous phase into intermetallic compounds, such as Al_19_Ni_5_Dy_3_, DyAl_3_, and Al_9_Co_2_. Careful adjustment of the sintering temperature is important to achieve fully dense specimens and appropriate phase selection by *in-situ* crystallization of the amorphous Al-Ni-Co-Dy alloy. The solute concentration plays a key role in determining the size of the α-Al phase during SPS of amorphous Al_84_Ni_7_Co_3_Dy_6_ powder. Al-Ni-Co-Dy bulk specimens SPSed at a sintering temperature of 673 K exhibit a maximum strength of 1773 MPa with 5.6% plastic strain. Larger values of plastic deformability of up to 7.2% can be achieved for sintering at 703 K, but at the expense of a lower maximum strength (1255 MPa). The present findings reveal that high strength and ductile Al-base alloys can be obtained through appropriately adjusted SPS treatment of gas atomized amorphous powders. This opens new perspectives for developing high strength Al-base alloys for high performance applications.

## Methods

Alloy ingots with nominal composition of Al_84_Ni_7_Co_3_RE_6_ (RE: Y, Gd, Dy) (at%) were prepared by arc melting under Ar atmosphere (purity 99.99%). To improve the homogeneity of the alloy buttons, they were re-melted four times. Typical mass losses of the samples were less than 1% of the initial mass. The arc-melted samples were crushed into small pieces and inductively re-melted in a fused silica tube, followed by ejecting with an over pressure of 35 kPa through a nozzle onto a rotating copper wheel of melt spinning equipment (Samhan vacuum SV-RSP202) with a surface velocity of 40 m/s to obtain amorphous ribbons of the alloys. The resulting ribbon samples had a thickness of about 20 μm and a width of about 3 mm. Al-Ni-Co-Dy amorphous powder was produced by high pressure gas atomization and the amorphous powder particles with a size of under 25 μm was used for consolidation into bulk samples with 20 mm diameter and 10 mm height by spark plasma sintering (SPS) with tungsten carbide die [SCM SPS-1050, heating rate 100 °C/min, pressure 500 MPa, time 5~7 min] The thermal stability of the samples was studied by differential scanning calorimetry (DSC) [TA Instruments DSC-Q100] in isochronal and isothermal modes. The isochronal DSC scans were performed at different heating rates of 5, 10, 20, 40 and 80 K/min. The isothermal DSC studies were carried out at annealing temperatures of 528, 533, 538 and 543 K. Structural characterization was done by X-ray diffraction (XRD) with monochromatic Cu Kα radiation [Model D/Max 2500PC Rigaku] and transmission electron microscopy (TEM) [JEM-2001F]. Thin foil samples for TEM analysis were prepared by conventional ion milling using a Gatan Model 691 PIPS (Gatan, USA). The parameters of the glass-crystal transformation kinetics were estimated by the Kissinger method^38,39^ and the Johnson-Mehl-Avrami (JMA) equation^29–31^. The mechanical properties of consolidated specimens were evaluated by room temperature compression tests (Hounsfield TX0056-H25KT). The SPSed samples were cut and polished to produce 2 mm wide and 2 mm thick specimens with a 4 mm gauge length. The compression tests were conducted at room temperature at a strain rate of 10^−4^ s^−1^.

## Electronic supplementary material

Supplementary Information
