# Supplementary material for: High strength nanostructured Al-based alloys through optimized processing of rapidly quenched amorphous precursors
Source: Sci Rep. 2018 Jan 18;8:1090. doi: 10.1038/s41598-018-19337-7 (PMC5773607; doi:10.1038/s41598-018-19337-7)
Supplement: Supplementary file 1 — Supplementary Information [file 41598_2018_19337_MOESM1_ESM.doc]

**Supplement**

**High strength nanostructured Al-based alloys through optimized processing of rapidly quenched amorphous precursors**

Song-Yi Kim^1*^, Gwang-Yeob Lee^2,3*^, Gyu-Hyeon Park^4^, Hyun-A Kim^1,3^, A-Young Lee^1^, Sergio Scudino^5^, Konda Gokuldoss Prashanth^6,7^, Do-Hyang Kim^3^, Jürgen Eckert^6,8^ and Min-Ha Lee1,[[1]](#footnote-2)

**^1^**Advanced Functional Materials R&D Group, Korea Institute of Industrial Technology, Incheon 21999, Korea

**^2^**Advanced Analysis Center, Korea Institute of Science and Technology, Seoul 02792, Korea

**^3^**Deparment of Advanced Materials Engineering, Yonsei University, Seoul 03722, Korea

**^4^**IFW Dresden, Institute for Metallic Materials, Helmholtzstraße 20, D-01069 Dresden, Germany

**^5^**IFW Dresden, Institute for Complex Materials, Helmholtzstraße 20, D-01069 Dresden, Germany

^6^Erich Schmid Institute of Materials Science, Austrian Academy of Sciences, Jahnstraße 10, A-8700 Leoben, Austria

**^7^**Norwegian University of Science and Technology, Teknologivegen 22, 2815, Gjøvik, Norway

**^8^**Department Materials Physics, Montanuniversität Leoben, Jahnstraße 10, A-8700 Leoben, Austria

Table S1 Characteristic thermal stability data (heating rate 40 K/min) of amorphous Al-Ni-Co-RE (Y, Gd, Dy) melt-spun ribbons.

| Composition | T_g_ (K) | T_x1_ (K) | T_x2_ (K) | T_x3_ (K) | ΔT (K) | ΔH_1_ (J/g) | ΔH_2_ (J/g) | ΔH_3_ (J/g) | Total ΔH  (J/g) |
| --- | --- | --- | --- | --- | --- | --- | --- | --- | --- |
| Al_84_Ni_7_Co_3_Y_6_ | 545±1 | 564±1 | 617±1 | 659±1 | 19±1 | 55.7±0.5 | 44.5±0.5 | 64.3±0.5 | 164.5±0.5 |
| Al_84_Ni_7_Co_3_Gd_6_ | 558±1 | 576±1 | 618±1 | 661±1 | 18±1 | 57.7±0.5 | 18.8±0.5 | 54.3±0.5 | 130.8±0.5 |
| Al_84_Ni_7_Co_3_Dy_6_ | 561±1 | 579±1 | 619±1 | 661±1 | 18±1 | 58.8±0.5 | 22.3±0.5 | 63.7±0.5 | 144.8±0.5 |

S1. (a) DSC traces obtained during continuous heating of as-spun Al_84_Ni_7_Co_3_RE_6_ (RE: Y, Gd, Dy) ribbons at a heating rate of 40 K/min (b) XRD patterns of as-spun Al_84_Ni_7_Co_3_Dy_6_ ribbons heated to 578 K, 618 K, 667 K and 773 K, respectively.

(b)

(a)

1. *These authors contributed equally to this work and S. Y. Kim and G. W. Lee are co-first authors

   ? Corresponding author: Tel.: +82 32 850 0424 ; fax: +82 32 850 0304; e-mail address: [mhlee1@kitech.re.kr](mailto:mhlee1@kitech.re.kr) (Lee MH) [↑](#footnote-ref-2)
